# Relationship between Retinal Microvasculature, Cardiovascular Risk and Silent Brain Infarction in Hypertensive Patients

**DOI:** 10.3390/diagnostics11060937

**Published:** 2021-05-24

**Authors:** Rosa Forés, Josep M. Manresa, Victor M. López-Lifante, Antonio Heras, Pilar Delgado, Xose Vázquez, Susana Ruiz, Maria Teresa Alzamora, Pere Toran

**Affiliations:** 1 Unitat de Suport a la Recerca Metropolitana Nord, Institut Universitari d’Investigació en Atenció Primària Jordi Gol (IDIAP Jordi Gol), 08303 Mataró, Spain; jmmanresa@idiapjgol.info (J.M.M.); vmlopezli.mn.ics@gencat.cat (V.M.L.-L.); aheras.bnm.ics@gencat.cat (A.H.); maiteal2007@gmail.com (M.T.A.); ptoran.bnm.ics@gencat.cat (P.T.); 2 Riu Nord-Riu Sud Primary Healthcare Centre, Santa Coloma de Gramenet, Gerència d’Àmbit d’Atenció Primària Metropolitana Nord, Institut Català de la Salut, 08921 Barcelona, Spain; 3 Palau Healthcare Centre, Palau-Solità Plegamans, Gerència d’Àmbit d’Atenció Primària Metropolitana Nord, Institut Català de la Salut, 08124 Barcelona, Spain; 4 Neurovascular Research Lab, Vall D’Hebron Research Institute, Universitat Autònoma de Barcelona, 08035 Barcelona, Spain; pilar.delgado@vhir.org; 5 Dementia Unit, Neurology Service, Vall D’Hebron University Hospital, Universitat Autònoma de Barcelona, 08035 Barcelona, Spain; 6 Hospital Municipal de Badalona, Badalona Serveis Assistencials, Secció d’Oftalmologia, Badalona, 08911 Barcelona, Spain; xvazquez@bsa.cat; 7 Germans Trias i Pujol University Hospital, Badalona, 08916 Barcelona, Spain; sruizbilbao@gmail.com

**Keywords:** silent brain infarction, cardiovascular risk, hypertension, retinal microvasculature

## Abstract

Objective: The aims of this study are to analyze the role of artery-vein ratio AVR assessment using VesselMap 2 software (Imedos Systems) and cardiovascular risk evaluation by means of REGICOR in the prediction of silent brain infarction (SBI) in middle-age hypertensive patients from the ISSYS study. Material and Methods: A cross-sectional study with 695 patients with hypertension aged 50 to 70 years who participated in the project *Investigating Silent Strokes in HYpertensives: a Magnetic Resonance Imaging Study* (ISSYS), was conducted in two Primary Care Centres of Barcelona. Participants agreed to a retinography and an MRI to detect silent brain infarction (SBI). The IMEDOS software was used for the semiautomatic caliber measurement of retinal arteries and veins, and the AVR was considered abnormal when <0.66. The REGICOR score was calculated for all patients. Results: Multivariate logistic regression analysis was used to evaluate the impact of AVR and REGICOR scores on SBI. The OR (odds ratio) for a high REGICOR score and an abnormal AVR were 3.16 and 4.45, respectively. When analysing the interaction of both factors, the OR of an abnormal AVR and moderate REGICOR score was 3.27, whereas with a high REGICOR score it reached 13.07. Conclusions: The measurement of AVR in patients with hypertension and with a high REGICOR score can contribute to the detection of silent brain infarction.

## 1. Introduction

Silent brain infarction (SBI) is a brain injury diagnosed with magnetic resonance imaging (MRI). Initially described by Fisher [1], epidemiological evidence shows the association between SBI and cognitive dysfunction [2], dementia [3], symptomatic stroke, and increased overall mortality [4]. A study by Gupta and colleagues [5] suggests that one in five adults over 60 with no history of stroke may have SBI, and SBI increases the risk of stroke more than twofold.

The factors most strongly associated with SBI are age, hypertension (HT), carotid stenosis, chronic kidney disease, and metabolic syndrome [6]. In turn, HT is a cardiovascular risk factor associated with increased risk of heart disease, encephalopathy, and kidney disease [7]. The WHO estimates a global prevalence of 1.13 billion people with hypertension, one of the main causes of premature death. In 2015, one in four men and one in five women had hypertension [7]. Menéndez et al. observed in 2016 that 42.6% (49.9% men and 37.1% women) of the Spanish population aged 18 years and over was hypertensive [8]. Recent publications address the effect of hypertension on the retinal microvasculature [9,10], with studies linking milder degrees of retinopathy and abnormal caliber of retinal vessels with increased risk of stroke [11,12,13], heart disease [14], stroke and heart disease [15], and cognitive decline [16,17].

Decreasing the incidence of cardiovascular disease remains a public health priority. Different scales adapted to specific populations have been created to assess cardiovascular risk [18,19]. The REGICOR scale, the most used in our setting, is a validation of the FRAMINGHAM [20] scale adapted to the Mediterranean population. The REGICOR scale [18] includes the following variables: age, sex, smoking, diabetes, total cholesterol, HDL cholesterol, and systolic and diastolic blood pressure. 

While fundoscopy has been routinely used to screen for diabetic retinopathy in patients with diabetes, patients with hypertension are not regularly screened for hypertensive retinopathy. However, innovative computer software has already been developed to semi-automatically interpret eye fundus lesions and to measure the retinal microvasculature. This program will facilitate the use of fundoscopy to evaluate patients with hypertension [21,22,23,24] and to screen for asymptomatic cardiovascular disease [25]. One measurement used is the retinal artery-vein ratio (AVR), which calculates the ratio between the mean arteriolar and venular width measured in the area of interest around the optic disc. 

The aims of this study are to analyze the role of AVR assessment using VesselMap 2 software (Imedos Systems GmbH, Jena, Germany) and cardiovascular risk evaluation by means of REGICOR in the prediction of SBI in middle-age hypertensive patients from the ISSYS study.

## 2. Methods

The study subjects were participants of Investigating Silent Strokes in Hypertensives: a Magnetic Resonance Imaging Study (ISSYS) [26], where the methodology of the study is described. The ISSYS study was conducted on a randomized sample of patients with hypertension between the ages of 50 and 70 usually managed in their primary care center, to determine the prevalence of SBI. Patients were randomly selected from a sample of 27,000 subjects living in the northern areas of Barcelona after stratifying by age and sex. Participants received a phone call inviting them to a visit where study researchers evaluated eligibility [26]. 

Inclusion criteria: patients diagnosed with hypertension for at least one year; age between 50 and 70 years; informed consent to perform a retinography. 

Exclusion criteria: prior history of stroke or dementia; contraindication to performing MRI; suspected white coat hypertension; terminal illness; anticipated difficulties for follow up; physical and functional limitation to obtain and interpret the retinography: media opacity, retinal photocoagulation, myopia magna, functional single eye and eye movement disorders. 

### 2.1. Procedure

During the ISSYS project visit, subjects were asked to undergo a retinography examination in one of the two designated Primary Care Centers between March 2011 and July 2012. Each participant underwent a retinography of both eyes centered on the optic disc/macula, at 45º, without mydriasis and under similar light conditions. Papilla-centered images were also taken when possible. A TOPCON TRC-NW65 retinograph was used, which consisted of the following: a Nikon D7000 camera for TRC-NW6, IbaseDigiCaptura, a 19” TFT monitor, computer with Windows 7 operating system, type I, ATE-600, and the VesselMap 2 software (Version 3.10) (Imedos Systems GmbH, Jena, Germany) to visualize and measure the retinal vasculature images (developed by IMEDOS Systems). The images were stored in jpg format on a purpose-built server.

**Figure 1:**
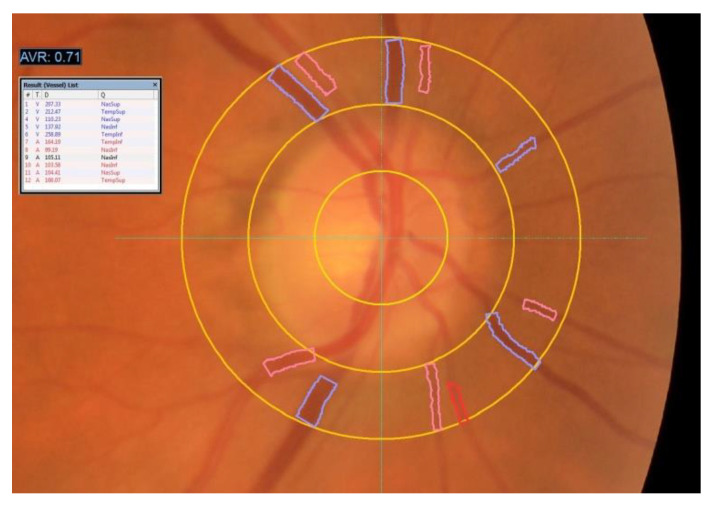
Semi-automatic measurement of artery and vein diameters by the VesselMap software. https://imedos.com/en/products/sva/ (accessed on 16 April 2021).

Two experienced ophthalmologists evaluated the retinographies and recorded the following lesions: abnormal AVR, abnormal arteriovenous crossings, microaneurysms, flame hemorrhages, soft exudates, hard exudates, and papilledema. The subjective ophthalmoscopic AVR measurement was replaced by the semiautomatic caliber measurement of arteries and veins by the VesselMap software, made by a technician prior to the training (Figure 1). With this system, the vessels are manually located and the AVR calculation is automatically provided. Values <0.66 are considered abnormal [27,28].

### 2.2. Statistical Analysis

Qualitative variables are described with absolute frequencies and percentages, while mean and standard deviation were used to describe continuous variables. Pearson’s Chi-square test was used for comparison of qualitative variables, and Student’s t-test for comparison of continuous variables. We conducted a multivariate logistic regression analysis where SBI was the dependent variable while abnormal AVR, REGICOR Risk and the combination of both were the independent factors. The level of significance was 5%. For all analyses, we used the SPSS statistical package for Windows, version 26.0.

## 3. Results

**Figure 2:**
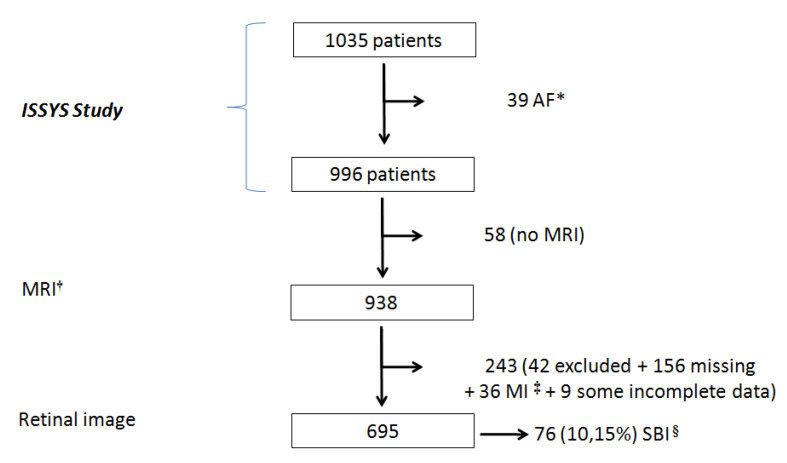
Flow Chart. * AF: Atrial Fibrillation; † MRI: Magnetic Resonance Imaging; ‡ MI: Myocardial Infarction; § SBI: Silent Brain Infarction.

Figure 2 shows that from the initial 1035 patients, 39 were excluded due to atrial fibrillation, 58 because the MRI could not be performed, and 243 because we could not locate them, their refusal to undergo the retinography, and other exclusion criteria. Finally, the retinography was performed on 695 participants and SBI was diagnosed in 76 (prevalence of 10.15%).

Table 1 describes participants’ characteristics based on the absence or presence of SBI. Of these characteristics, we should underscore the following four: REGICOR > 10 in 20.7% and 34.3% (*p* = 0.014); AVR < 0.66 in 12.7% and 38.8% (*p* < 0.001) in patients without and with SBI, respectively.

A multivariate logistic regression analysis was performed to calculate the risk of SBI with abnormal AVR and the REGICOR risk score (Table 2A). The results show an Odds Ratio of 3.16 with a high REGICOR score and of 4.45 with an abnormal AVR. In the model with the interaction of both factors, an abnormal AVR plus a moderate REGICOR risk resulted in an Odds Ratio of 3.27, while with an abnormal AVR plus a high REGICOR risk the Odds Ratio increased to 13.07 (Table 2B).

## 4. Discussion

We believe that the results of this study can be extrapolated to the population between 50 and 70 years of age with hypertension in our setting, because the ISSYS study is a randomized, population-based study and the baseline characteristics of participants who underwent and did not undergo retinography did not significantly differ. [29]

SBI prevalence in our study was over 10%, a value in the lowest range amongst population-based epidemiological studies [6], and lower than the prevalence described in cohorts of patients with hypertension. These differences could be attributed to the younger average age and better control of hypertension in the current cohort [29].

Our findings corroborate results observed in other population-based studies, [11] which suggest that hypertensive retinopathy is an independent cardiovascular risk factor. Yatsuda et al. [12] observed that the narrowing of the central artery and widened central retinal vein caliber, focal arteriolar narrowing, and arteriovenous crossings were predictors of lacunar stroke in a cohort of adults followed for an average of 11 years. In a meta-analysis by Dumitrascu et al., [30] venular dilation was associated with the appearance of lacunar stroke. Our study shows that the semi-automatic measurement of AVR < 0.66 correlates better with SBI than any other signs of hypertensive retinopathy, possibly thanks to the removal of the observer’s subjectivity [31]. 

Ikram et al. [32] describe the association between widened retinal venules and the progression of microvascular brain lesions in a general population over 55 years of age. However, this association fades when retinal circulation is measured with the AVR. Subsequent studies that use automated vessel caliber measurement indicate that even in adolescents, hypertension has a greater impact on arterioles than on venules [33]. Most studies agree that due to as yet poorly defined underlying mechanisms, abnormalities in arterioles and venules are associated with different cardiometabolic risk factors: narrower arterioles with higher blood pressure; wider venules with atherosclerosis, inflammation, higher cholesterol levels, and higher body mass index [34].

Other factors to consider are gender, since arteriolar and venular diameters are smaller in women than men, and aging, which decreases the density of retinal vessels, reduces the thickness of the inner retinal layer and the speed of retinal blood flow, particularly in the venules [28]. In this study we could not evaluate the effect of the different retinopathy lesions such as crossings and microaneurysms, which were detected in low numbers. Furthermore, the semiautomatic reading data produced by the VesselMap software does not discriminate between the diameters of the arteries and small veins to elucidate the most significant components in the variation of the AVR. However, with the addition of the REGICOR score to the AVR, we incorporated factors known to affect the diameter of arterioles and venules such as age, sex, blood pressure, and cholesterol.

The findings of this study suggest that AVR measurements in patients with hypertension are useful to screen people at high risk of SBI, particularly in patients with high REGICOR risk. To this end, the automation of retinal vasculature measurements during retinography remains crucial.

In conclusion, we recommend retinography screening to measure the AVR in all patients with hypertension and a REGICOR score >10 and to evaluate the possibility of carrying out a MRI for the detection of SBI. The diagnosis of SBI would streamline treatment and follow-up, particularly the prevention of symptomatic stroke in this high-risk group of patients. 

## Figures and Tables

**Table 1:** Description of characteristics of the 695 participants based on the presence or absence of SBI.

	NO SBI *	SBI	TOTAL	
	N = 628	N = 67	N = 695	*p*
Age	62.4 (5.6)	64.6 (4.7)	62.7 (5.6)	0.002
Men	285 (45.4%)	49 (73.1%)	334 (48.1%)	0.000
Former smoker	94 (15.0%)	11 (16.4%)	105 (15.1%)	0.753
Dyslipidemia ^†^	438 (70.1%)	52 (78.8%)	490 (70.9%)	0.139
REGICOR				0.014
Low < 5	170 (27.1%)	10 (14.9%)	180 (25.9%)	
Moderate 5–10	328 (52.2%)	34 (50.7%)	362 (52.1%)	
High > 10	130 (20.7%)	23 (34.3%)	153 (22.0%)	
**Detected Alterations in Retinography**				
AVR ^‡^ < 0.66	80 (12.7%)	26 (38.8%)	106 (15.3%)	0.000
Arteriovenous nicking	407 (64.8%)	45 (67.2%)	452 (65.0%)	0.701
Mycroaneurysms	14 (2.2%)	3 (4.5%)	17 (2.4%)	0.258
Flame hemorrhages	0 (0.0%)	1 (1.5%)	1 (0.1%)	---
Soft exudades	6 (1.0%)	0 (0.0%)	6 (0.9%)	0.422
Hard exudates	6 (1.0%)	2 (3.0%)	8 (1.2%)	0.39

* SBI: Silent Brain I* SBI: Silent Brain Infarction; † Dyslipidemia: 3 + 1 missings; ‡ AVR: Artery-Vein Ratio.

**Table 2:** Model obtained from the multivariate logistic regression analysis for the risk of presenting silent cerebral infarct.

A-Adjusted Model	β Coefficient	OR (CI 95%) ^a^	*p*
Constant	−3.252		
**REGICOR**			
Low < 5	Reference		
Moderate 5–10	0.652	1.92 (0.91–4.04)	0.086
High > 10	1.150	3.16 (1.3–7.00)	0.005
**RAV < 0.66** ^b,c^	1.493	4.45 (2.56–7.73)	<0.001
**B-Interaction Model**	**β Coefficient**	**OR (CI 95%) ^a^**	***p***
Constant	−2.570		
AVR > 0.66 + Low REGICOR	Reference		
AVR < 0.66 + Moderate REGICOR	1.184	3.27 (1.53–6.97)	0.002
AVR <0.66 + High REGICOR	2.570	13.07 (5.71–29.90)	<0.001

CI 95%: Confidence Interval 95%; ^a^ Dependent Variable: Silent brain infarction (diagnosed by MRI); ^b^ Abnormal AVR when <0.66; ^c^ AVR obtained with the Vesselmaps2 software version 3.10 (Imedos).

## Data Availability

The data presented in this study are available on request from the corresponding author.
